# Systematic Comparison of Brain Imaging Meta-Analyses of ToM with vPT

**DOI:** 10.1155/2017/6875850

**Published:** 2017-03-07

**Authors:** Aditi Arora, Matthias Schurz, Josef Perner

**Affiliations:** Centre for Cognitive Neuroscience, University of Salzburg, Hellbrunnerstr. 34, 5020 Salzburg, Austria

## Abstract

In visual perspective taking (vPT) one has to concern oneself with what other people see and how they see it. Since seeing is a mental state, developmental studies have discussed vPT within the domain of “theory of mind (ToM)” but imaging studies have not treated it as such. Based on earlier results from several meta-analyses, we tested for the overlap of visual perspective taking studies with 6 different kinds of ToM studies: false belief, trait judgments, strategic games, social animations, mind in the eyes, and rational actions. Joint activation was observed between the vPT task and some kinds of ToM tasks in regions involving the left temporoparietal junction (TPJ), anterior precuneus, left middle occipital gyrus/extrastriate body area (EBA), and the left inferior frontal and precentral gyrus. Importantly, no overlap activation was found for the vPT tasks with the joint core of all six kinds of ToM tasks. This raises the important question of what the common denominator of all tasks that fall under the label of “theory of mind” is supposed to be if visual perspective taking is not one of them.

## 1. Introduction

The ability to see the world from another person's perspective and taking into account what they see and how they see it is called visual perspective taking (vPT). Visual perspective taking has played a central role in developmental investigations of children's understanding of other minds (Three Mountain Problem by Piaget and Inhelder [1]; its refinements by Flavell [2]; Flavell et al. [3]; Masangkay et al. [4]). Moreover, “seeing” is clearly a mental state as it involves subjective inner experience. Theory of mind (ToM) is defined as the ability to attribute and reason about mental states [5–8]. Hence, by definition, processing of what people see and of how they see it is part of ToM. Yet, in the brain imaging literature they are almost exclusively treated as independent fields of investigation. An early study looking for overlap in vPT and ToM processing [9] found only one small region in the left temporoparietal junction (TPJ)—liberally interpreted. A recent meta-analysis [10] of studies of false belief, a central concept of ToM [11–13], and vPT confirmed that overlap between these areas is sparse, consisting of small regions around the posterior part of left TPJ and of the precuneus.

Theory of mind consists, however, of more than just processing information about false beliefs. Schurz et al. [14] showed in a meta-analysis of six kinds of ToM tasks (false belief, trait judgments, strategic games, social animations, mind in the eyes, and rational actions) that they all activate core areas consisting of the left and right TPJ and the medial prefrontal cortex (mPFC). The meta-analysis showed marked differences in activation across the six subtypes of theory of mind tasks. An interesting question is with which of these tasks vPT can be expected to share a common process. We can make predictions based on various proposals in the literature about which aspects of theory of mind tasks are responsible for activation in particular brain regions. Those theory of mind tasks that share a particular feature in question with vPT should show activation overlap in the region claimed for that feature. In order to make such predictions we need to look at the central features of vPT tasks that entered the meta-analysis.

One potentially problematic shortcoming of the vPT meta-analysis is that there are not enough studies to date to warrant a separation between level 1 and level 2 perspective tasks. Level 1 perspective taking refers to the ability to judge what someone else can and cannot see; for example, two people looking at a piece of paper from opposite sides can see different things. Level 2 perspective taking requires understanding that two persons looking at the same array of objects from different viewpoints can arrive at different and sometimes contradictory descriptions. This distinction plays an important role in development. Level 1 tasks are mastered as early as 2–2.5 years [15–17], although an implicit level 1 ability may be developed in the 2nd year [18, 19]. Level 2 tasks are mastered around 4 years (Flavell et al., 1971) when children pass the standard verbal false belief task (there is a growing literature showing much earlier sensitivity to people's beliefs (e.g., Onishi & Baillargeon 2005; Southgate et al. 2007; Kovacs et al. 2010) followed by controversies of replicability (e.g., Phillips et al. 2015) and interpretation (e.g., [13, chapter 8]; Ruffman 2015)) [20]. Recent study by Moll and Meltzolf [21] has reported earlier development of level 2 perspective taking around the age of 3 years. Also children with autism who are known to have problems with theory of mind also have problems with level 2 perspective tasks [20] but not with level 1 tasks (e.g., [22]). Samson and colleagues [23, 24] have shown that level 1 tasks trigger automatically implicit perspective taking of the avatar, which is shown in prolonged reaction times when describing one's own view (altercentric intrusion) while level 2 tasks do not show this effect [25]. The spontaneous computation of level 1 perspective has been investigated with behavioural studies. However, recently Ramsey et al. [26] have also provided brain based evidence that other people's perspectives are automatically computed and no cognitive control is required to select another's perspective over one's own. Recent studies have debated the automatic computation of level 1 perspective. Santiesteban and colleagues observed prolonged reaction time even in experiments where the avatar was replaced by an arrow, suggesting that arrow cues have the same attention orienting effect as does the avatar [27, 28]. Modifications of the dot perspective task (such as use of transparent and opaque barriers or the use of transparent and opaque goggles) yielded results inconsistent with spontaneous perspective taking [29]. Though, Furlanetto et al. [30] using the dot perspective task demonstrated that the consistency effect occurred only when the avatar wore the transparent goggles but not for the opaque goggles. This supports the automatic mentalizing interpretation. Conway et al. (2016) considered the possibility that the difference between the Cole and Furlanetto studies could be due to methodological differences.

Nevertheless, when asking directly about what another person can see, level 1 tasks are likely to also trigger level 2 processes, even though they are not strictly speaking required for the task. For instance, when asked the level 1 question of how many objects another person can see, one can get away with simply reporting how many objects are within his visual field without concern about how these objects and their arrangement might look to him (level 2). For this reason children find level 1 tasks much easier. But adults might spontaneously concern themselves with the appearance of the scene for the other person. For this reason we checked whether the level 1 and level 2 tasks in the meta-analysis tended to activate particular regions differentially. There is no observable difference between tasks (see Table 3 in [10]), and the majority of tasks in the meta-analysis were clear cases of level 2 tasks in any case.

## 2. Open Questions about vPT and Theory of Mind Overlap

Our study addresses two important questions about the relationship between vPT and ToM. Of central concern is the question whether vPT overlaps with the core areas of ToM. Since Schurz et al. [10] found no overlap of vPT with false belief vignettes except in the left IPL, we can exclude the possibility that there will be overlap in all three core areas reported by Schurz et al. [14]. Nevertheless, vPT might overlap with the core area in the left TPJ. Hence on the basis of the interpretation by Schurz et al. that the core area is responsible for attributing mental states, we expect overlap in this region.

The other interesting question is whether vPT shares brain activation with specific ToM tasks. This allows us to assess different proposals in the literature about where specific subprocesses of ToM should take place.  Table 1 gives a concise overview of these predictions.

## 3. Prediction (from General to Specific)

(1) Amodio and Frith [31] suggested that the anterior-rostral subregion of the medial prefrontal cortex (arMFC) is responsible for all reflection about mental states. This claim is supported by the theory of mind meta-analysis [14] which shows an overlap of all six kinds of theory of mind tasks in roughly this subregion of mPFC. Since vPT also requires reflection on a mental state of seeing, vPT should show activation overlap with ToM in this region.

(2) Gallagher and Frith [39] proposed that the medial prefrontal cortex (mPFC) is responsible for decoupling (Leslie, 1987) representations from primary representations depicting the actual state of the world. Level 1 vPT would not require such decoupling because in level 1 task three of the five objects in the room are within the avatar's visual field, representing the actual state of the world, whereas level 2 vPT contrast would require decoupling. To represent that he sees the three objects, for example, forming a triangle, while I see them in a row, requires decoupling, since these descriptions cannot simply be taken to represent different actual arrangements of the three objects but as two different views of the objects. To the degree that the vPT meta-analysis captures level 2 processes the prediction would be that vPT should show overlap with theory of mind in mPFC.

(3) Schurz et al. [14] found a core area of TPJ bilateral and mPFC shared by six kinds of theory of mind tasks. The authors suggested that this area is responsible for attributing mental states as the common denominator for all six kinds of theory of mind tasks. In this case we should find that vPT also activates this common area since vPT tasks require judgments of another person's mental state of seeing.

(4) A meta-analysis by Van Overwalle [32] showed that attribution of more enduring states activates mPFC while attribution of more transient mental states activates TPJ. Given this view vPT should activate TPJ but not mPFC.

(5) Gobbini et al. [33] generalized from their study that ToM tasks in which one can perceive action in movements activate more ventral parts of the temporoparietal junction (TPJ/pSTS) than tasks in which mental states or actions are only verbally described, which activate TPJ/IPL (FB vignettes). In vPT tasks no overt action is observed. Hence vPT should not activate TPJ/pSTS. Also no verbal descriptions are used but one sees what another person sees. Hence vPT should not activate TPJ/IPL either.

(6) Mar [34] found in a meta-analysis more dorsal activation in the TPJ (TPJ/IPL) for story-based ToM tasks than for non-story-based (and largely nonverbal) ToM tasks. The vPT tasks do not involve stories but require judgments of visually presented situations. Hence we expect no overlap in TPJ/IPL.

(7) Saxe et al. [35] proposed that the right temporoparietal junction (rTPJ) is responsible for processing information about mental states with propositional content, in particular thoughts and beliefs. If we take propositional content as the critical feature then level 1 vPT (e.g., he sees three objects) should not show an overlap in this region, but level 2 vPT should do so (he sees the three objects forming a triangle). In the understanding that vPT tasks engage level 2 processes then vPT should activate the rTPJ. If the critical criterion is attribution of thoughts and beliefs then clearly visual perspective taking need not activate this region.

(8) Perner and Leekam [36] observed that studies that involve perspective differences (e.g., false belief vignettes) activate more dorsal parts of the TPJ (TPJ/IPL) than studies in which perspective differences play no role (e.g., intentional actions). This separation is particularly pronounced in the left TPJ. Here we expect vPT to activate the left TPJ/IPL, definitely if level 2 processes are involved.

(9) C. D. Frith and U. Frith [37] suggested that the posterior superior temporal sulcus (pSTS) is activated by information about a person's action. Based on this account vPT should not activate the pSTS.

(10) Samson and colleagues (e.g., [38, 40]) presented accumulating evidence from behavioural and patient studies which suggests that multiple neurocognitive mechanisms contribute to ToM. Representing mental states like beliefs engages the left TPJ and (possibly also) the mPFC [41, 42], whereas inhibiting one's self-perspective in mentalizing tasks engages the right lateral frontal cortex and in particular the right IFG [43, 44]. Moreover, it was found in a vPT task that the lateral prefrontal cortex is engaged whenever a task-relevant perspective needs to be selected over an irrelevant one, irrespective of whether it is the perspective of self or other [26]. Since vPT tasks require representation of the mental state of seeing and suppression of one's own perspective, vPT should overlap with ToM in the left TPJ and in the right IFG.

## 4. Method

To study the components of vPT in a ToM task, we considered the vPT meta-analysis by Schurz et al. [10]. Their vPT meta-analysis included 14 studies (*N* = 216), including both level 1 and level 2 visual perspective taking studies to increase the statistical power of the vPT meta-analysis (see Table 2, for examples of included studies). For ToM tasks, we considered a meta-analysis by Schurz et al. [14] that included six different task groups (see Table 3, for examples from each task-group): (i) false belief; 15 studies (*N* = 259), (ii) trait judgment; 15 studies (*N* = 253), (iii) strategic game; 9 studies (*N* = 162), (iv) social animation; 14 studies (*N* = 224), (v) mind in the eyes; 10 studies (*N* = 185), and (vi) rational action; 10 studies (*N* = 158). Both meta-analyses were performed using Effect-Size Signed Differential Mapping (ES-SDM) software, version 2.31 for meta-analysis ([45, 46], http://www.sdmproject.com). For the conjunction analysis, vPT and ToM tasks' meta-analytic result maps (in Talairach space) were used, thresholded using a voxel-level (heights) threshold of *p* < 0.005 (uncorrected) and cluster-level (extent) threshold of 10 voxels. The conjunction analysis was performed in three steps using “image calculator” utility in SPM8 (http://www.fil.ion.ucl.ac.uk). First, a conjunction analysis was performed between the core regions activated in the ToM tasks, using the permutation based conjunction map from Schurz et al. [14] meta-analysis and the vPT meta-analytic map. A similar conjunction was then performed between the ToM pooled meta-analysis maps including all studies (*n* = 73) and the vPT meta-analysis. Second, to determine the components of vPT among the ToM tasks, we performed a conjoint activation analysis between the vPT task and separate ToM tasks using voxel-wise combination of results by a logical AND function. Third, to find the overlap between the different task sets (vPT and ToM) that tend to coactivate, we analyzed meta-analysis maps using the equation [(*i*_1_ + *i*_2_ + *i*_3_ + *i*_4_ + *i*_5_ + *i*_6_) > 0, 1, or 2] in the “image calculator,” where *i*_1_, *i*_2_, *i*_3_,… were conjunction images created at the second step and 0, 1, or 2 numbers after “>” sign were used to create conjoint maps among different task groups.

## 5. Results

**Figure 1:**
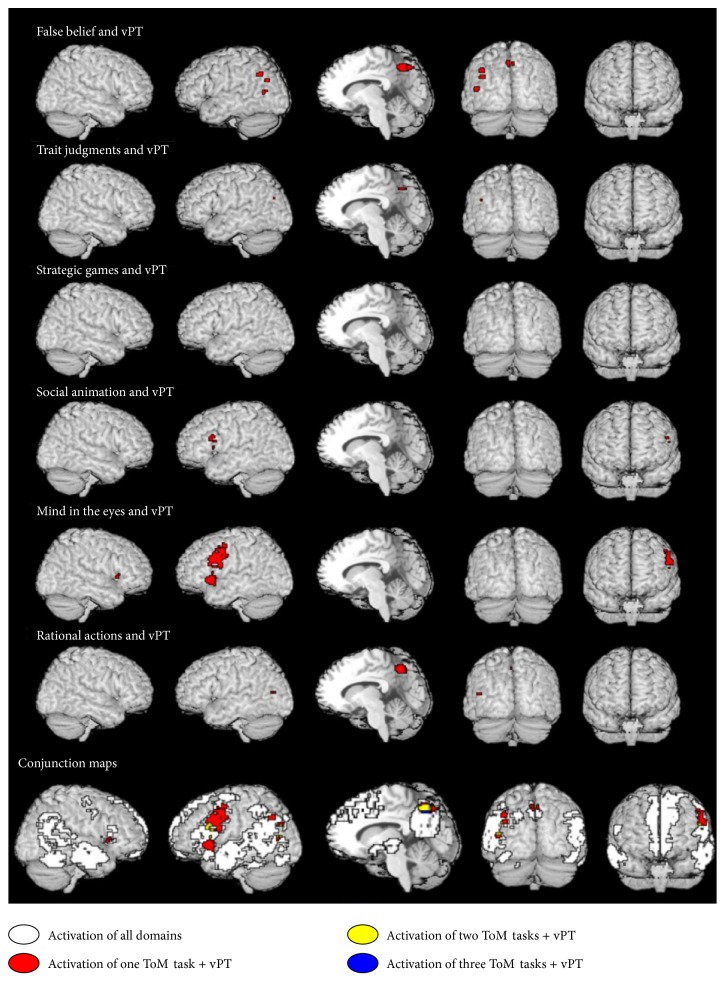
Conjunction maps of all theory of mind (ToM) meta-analyses and visual perspective taking (vPT). White indicates the regions activated by one meta-analysis; red and yellow indicate the conjunction of at least one and two meta-analysis along with vPT—see Table 4 for peak coordinates and overlap details. All meta-analytic maps were thresholded at voxel-wise threshold of *p* < 0.005 uncorrected and a cluster extent threshold of 10 voxels. Activation of all meta-analysis is superimposed on Talairach.

We performed a conjunction analysis between the vPT meta-analysis [10] and the ToM meta-analysis [14] to investigate the common components of vPT in ToM tasks. As proposed earlier, we looked for a conjunction of the ToM “core region” [14] with the regions in the vPT meta-analysis by Schurz et al. [10]. We did not observe any overlapping activation. However, an overlap was observed between the regions of the ToM pooled meta-analysis including all studies (*n* = 73) and regions of the vPT meta-analysis, showing common components between the two meta-analyses. In the next step, we wanted to see which particular ToM tasks contributed to this overlap. Thus, we performed a conjunction analysis for the vPT and each group of ToM tasks. This analysis showed overlapping activation in the left TPJ (angular gyrus) for vPT and false belief; in the posterior middle temporal gyrus for vPT, false belief and trait judgments; in the anterior precuneus for vPT, false belief, trait judgments, and rational actions; in the precentral gyrus, inferior frontal gyrus, and frontal operculum for vPT, social animations, and mind in the eyes; and in the left middle occipital gyrus for vPT, false belief, and rational actions (see Figure 1, Table 4). The potential functional role of these regions of overlapping activation is discussed in the following sections.

## 6. Discussion

We first discuss the predictions drawn from the suggestions about functional localisation in the literature. Then we look at each area of overlap in more detail.

Figure 1 and Table 4 make it clear that the lack of vPT activation in mPFC speaks against the suggestion of Amodio and Frith (2006) that this region is involved in reflection of mental states and also against Gallagher et al.'s (2003) proposal that mPFC is responsible for decoupling. It also refutes the conclusion from the meta-analysis by Schurz et al. [14] that mPFC is part of the core area of all mentalizing.

The fact that vPT only overlaps with some theory of mind tasks in the left TPJ/IPL but not in the right TPJ/IPL, nor in the TPJ/pSTS area, also poses a problem for several of the proposals. It speaks against the conclusion from the meta-analysis by Schurz et al. [14] that TPJ is bilaterally involved in all mental state attribution and not only its dorsal (TPJ/IPL) but also its more ventral (TPJ/pSTS) part. The lack of bilaterality of TPJ activation speaks against Van Overwalle's [32] claim that left and right TPJ are involved in attributing temporary mental states. The activation of TPJ/IPL without activation of TPJ/pSTS speaks against Gobbini et al.'s [33] proposal that the dorsal part of TPJ is responsible for covert mental states and its ventral part for mental states displayed in behaviour. The TPJ activation also speaks against Mar's [34] claim that dorsal TPJ is responsible for story-based theory of mind. The lack of overlap in right TPJ speaks against the wider interpretation of Saxe et al.'s [35] claim that right TPJ is responsible for all mental states with propositional content and it speaks in favour of the narrower claim that it is only responsible for attribution of beliefs and thoughts.

The data are compatible with the suggestion by Perner and Leekam [36] that left TPJ/IPL but not TPJ/pSTS is involved in perspective tasks, with C. D. Frith and U. Frith's [37], and Gobbini et al.'s proposal that pSTS is responsible for processing information about human actions. The data are also compatible with Samson and Apperly's [38] position that left TPJ is required for attributing mental states and that inhibiting one's own perspective requires the right inferior frontal gyrus (IFG). The data do, however, contradict their suggestion in terms of laterality. We did not find activation in the right but in the left IFG instead (we note, however, that Samson and Apperly's [38] findings are not incompatible with the involvement of the left IFG in addition to the right one. For example, Ramsey et al. [26] found activation in bilateral IFG (and dorsolateral PFC) for perspective-selection in a vPT task).

We now turn to discussing the involvement of the relevant brain areas in more detail.

## 7. Left Temporoparietal Junction and Mid. Temporal Gyrus

The TPJ characterized as a region in the cerebral cortex is considered part of the border between the temporal and parietal lobe, at the end of the Sylvian fissure. The TPJ is activated by a series of different experimental tasks like memory, attention, language, and social cognition. Interestingly, activation of temporoparietal areas by theory of mind tasks showed functionally distinct activation in dorsal and ventral parts of the left TPJ [7, 33, 55, 56]. Perner and Leekam [36] argued that tasks that involve understanding perspective differences activate specifically in the dorsal part of the left TPJ. This observation can explain the overlap between visual perspective taking (vPT), false belief, and trait judgments. Both false belief and vPT are essentially concerned with representing another person's differing perspective. For trait judgments this is not so obvious. Traits are based on habitual patterns of behaviour or thought. For instance, Mitchell et al. [57] showed participants photographs of males paired with personality trait statements, for example,* “he persists on his point in the meeting.”* Judging whether that person is stubborn requires the understanding that the person has a different perspective on the worth of his arguments compared to the normal person. Therefore, he persists in it when all others think it is hopeless. Since not all traits used in these experiments are traits based on perspective difference, the left dorsal TPJ activation is weaker than for false belief and vPT tasks. This shows a larger overlap between false belief and vPT than the overlap between vPT and trait judgments.

In the mind in the eyes task, participants have to judge the emotion shown in a person's eyes [53] and are not required to take the other persons' perspective. Hence, activation does not overlap with vPT in the left TPJ. Similarly social animation and rational action tasks did not show any overlap with vPT either, as these tasks do not require understanding of other persons' differing perspective. Rather, their activation can be explained by belief-desire reasoning [14, 36, 58, 59].

Finally, strategic games, the sixth kind of theory of mind paradigm included in Schurz et al.'s meta-analysis, are conspicuously absent in Table 4, since they showed no overlap with vPT. This may be due to the choice of contrast in these studies of playing against a person minus playing against a computer, which may subtract out interesting activation. Indeed, when Wieshofer (unpublished) looked at the baseline contrast for playing against another person she found activation in the left TPJ and in the anterior precuneus, supporting the view that these areas are involved in perspective taking.

## 8. Precuneus

There has been growing interest in the functional significance of the precuneus (Brodmann's area, BA 7). Even though precuneus activation is considered to be a robust neural correlate of ToM, its functional significance has not been explored much. In their review Cavanna and Trimble [60] suggested a functional distinction between anterior (*y* close to −60) and posterior (*y* close to −70) precuneus.

Anterior precuneus activation is typically observed during visuospatial imagery and motor imagery [61, 62], mental imagery during cognitive tasks like deductive reasoning [63, 64], episodic retrieval [65, 66], self-referential judgment [67], first-person perspective [68–70], and third-person perspective taking [71–73].

We observed that ToM tasks (false belief, trait judgment, and rational action) overlapped with vPT in the anterior region of precuneus. Cavanna and Trimble [60] proposed that the cognitive function of the anterior precuneus in first- and third-person perspective taking could be internal representation through mental imagery. This functional specification could account for the overlap among the four tasks. Each of them requires taking a third-person perspective and has to keep that perspective in mind by forming an image of it. The false belief task requires imagination to represent the protagonist's false belief. For at least some kinds of trait judgments one has to imagine a person's differing view, for example, the value of his arguments. Rational action tasks may require imagining the protagonist's goal (e.g., escaping from the cell) which is not shown in the pictures. Finally, vPT tasks may induce imagining the avatar's perspective, which is not required in the control task. To judge what one sees in oneself one does not have to form an image in addition to what one sees anyway.

However, a slight problem with Cavanna and Trimble's account arises from the fact that false belief activation is mostly taken from studies where the false belief condition was contrasted with the photo task. For instance (Aichhorn et al., 2009), the false belief vignette read “Julia sees the ice cream van go to the lake. She does not see that the van turns off to the town hall. Therefore, Julia will look for the ice cream van at the…Lake/Town Hall?” and the photo vignette “Julia takes a picture of the ice cream van in front of the pond. The ice cream van changes to the market place; the picture gets developed. In the picture the ice cream van is by the…Pond/Market place?” It is not clear why participants needed to form an image of Julia's belief, but not of Julia's photo in those tasks. The problem can be solved by paying close attention to Cavanna and Trimble's requirement that imagination activates anterior precuneus only in the case of representing someone's perspective (first or third person). This is clearly the case in the false belief vignette where one has to imagine the content of the belief as Julia's perspective of where the ice cream van is. This is not required in the photo vignette where one needs to imagine where the ice cream van is in the photo. One can treat the photo as an object in the world and need not treat it as giving a perspective on where the van was when the photo was taken.

Importantly, rational action tasks require imagining story characters' perspective, for example, what they intend to do, but there is no perspective difference involved as in the false belief vignettes. For this reason the rational action task activates the precuneus but not the left IPL.

Interestingly no precuneus overlap was observed for vPT with strategic game, social animation, or mind in the eyes tasks. The lack of precuneus activation of the strategic games may be due to the fact that all studies in the meta-analysis contrasted imagining playing against a human opponent versus a computer. Imagining the opponent's perspective should according to Cavanna and Trimble's criterion activate the precuneus. However, imagining the perspective of the computer as an opponent leaves no activation for the human > computer contrast. Indeed, when Wieshofer (unpublished) looked at the baseline contrast for playing another person she found activation in the anterior precuneus and the left TPJ showing that strategic games do require perspective taking. Social animation and mind in the eyes tasks do not activate the anterior precuneus in line with Cavanna and Trimble's functional specification because they do not require imagination beyond what one perceives. The moving figures in social animation create the perception of intentional interaction and mind in the eyes task shows the emotion of the person whose eyes one sees.

## 9. Left Mid. Occipital Gyrus/Extrastriate Body Area (EBA)

The overlap in the left middle occipital gyrus between the false belief, rational action, and visual perspective taking tasks was in good correspondence with the EBA coordinates (MNI coordinates: −51, −72, 8) reported by Downing et al. [74], who described this region as highly sensitive to the perception of human body and body parts, located in the posterior inferior temporal sulcus. Traditionally, the EBA was considered to be a region selective for visual processing of static images. Saxe and Wexler [75] reported a functional dissociation between the right and the left EBA. The right EBA preferentially responded to still photographs of body parts from an allocentric perspective, whereas the left EBA activity showed no difference between egocentric and allocentric perspectives to still photographs of body parts. Activation in the EBA was also observed when participants engaged in imagery of walking around the room [76]. Deen and McCarthy [77] reported activation in the EBA while participants were asked to read and comprehend vignettes involving statements about human biological motions.

The conjunction of neural activity during rational action, false belief, and visual perspective taking (vPT) tasks in the left EBA might therefore be due to two different processes. The rational action and the vPT task involve both static images of a human performing an action, which activates the left EBA [75]. The false belief task-related activity in the EBA, on the other hand, could be due to participants reading story vignettes about someone performing actions, which leads to EBA activation [77].

Consistent with our interpretation of the EBA and its function, no activation overlap was observed between the trait judgment, strategic game, social animation, and mind in the eyes tasks, as none of these tasks involved processing of imagined body movements, or reading vignettes describing human biological motion.

## 10. Left Inferior Frontal and Precentral Gyrus/Mirror Neuron System (MNS)

In addition to the parietal regions, activation overlap was also observed in the inferior frontal gyrus (IFG, involving the pars opercularis and pars triangularis which include Brodmann's areas 44 and 45) and the precentral gyrus among mind in the eyes, social animations, and the vPT tasks (see Figure 1, Table 4).

The common element of the overlapping activation in the IFG involves processing of biological cues: biological movement in the case of social animations, a facial expression in mind in the eyes tasks, and a person's eye gaze in the case of visual perspective taking. All three types of biological cues have been found to activate the IFG (e.g., biological movement: [78]; facial expressions: [79]; eye gaze: [80]). More specifically, social animations show geometrical shapes that move in a way that resembles key features of biological motion (i.e., movement appears self-propelled and contingent on the movement of other objects). For example, participants rate social animations as being more “animate” than their control conditions (e.g., [81]), which usually show mechanical movement patterns of the triangles (e.g., resembling the movement of billiard balls on the table).

In contrast, false belief, strategic games, and trait judgments did not show any overlap in the IFG or the precentral gyrus, which is consistent with the fact that those tasks do not present biological cues but more abstract stimuli.

In terms of cognitive processing, we speculate that social animations, mind in the eyes, and visual perspective taking could have in common the fact that they require mirroring functions. Biological motion is linked to mirror neurons in the context of action understanding (e.g., [82, 83]), recognition of facial expressions has been linked to mirror neurons in an emulation account (e.g., [79]), and eye gaze processing and gaze following have been linked to mirror neuron activity [84, 85].

## 11. Conclusion

In the introduction we raised two important questions to be investigated in this meta-analysis. One question was whether the vPT task activation overlaps with particular kinds of ToM tasks in different brain areas. The results enabled us to critically evaluate existing proposals in the literature and gain information about where component processes take place. We found activation overlap of the vPT task with false belief and trait judgments in the left TPJ and the posterior mid temporal gyrus, involved in perspectival thinking. In the anterior precuneus, overlap activation was observed between the false belief, trait judgment, and rational action tasks. Overlap with false belief and trait judgments are attributed to a common component of tracking another person's different perspective, whereas overlap with rational action is attributed to imagining another person's perspective. An overlap of false belief, rational action, and visual perspective taking tasks was found in the left mid occipital gyrus (EBA). These regions are implicated in motor imagery and human biological motion. The left IFG/precentral gyrus showed overlap between social animation, mind in the eyes, and the vPT tasks. This was explained by the coincidental activation of the mirror neuron system involved in execution and observation of action and empathy in the case of social animations and mind in the eyes and working memory in the case of vPT.

The other question was whether the vPT task activation overlaps with the core areas common to all types of ToM tasks. Schurz et al. [10] already established that there was no overlap with all three core areas but left open the possibility that in our complete meta-analysis with all six types of ToM tasks it might be possible to detect overlap in the left TPJ. Our results definitively rule out any overlap of vPT with any of the core ToM areas. This raises important issues about what exactly the common components of theory of mind tasks are. Schurz et al. [14, p 30] concluded from their meta-analysis “that the mPFC and bilateral posterior TPJ (connectivity cluster TPJp) showed activation for all theory of mind tasks. This is in line with claims about the existence of a ‘core-network' for theory of mind, that is, that all sorts of theory of mind tasks consistently engage a particular brain network (e.g., [8, 31]; Mitchell, 2009).” This conclusion is difficult to maintain in view of the lack of overlap with vPT task-related activation, since vPT involves attributing the mental state of seeing. Therefore, it might be more accurate to characterize the network, which is commonly activated by theory of mind tasks as a* belief-desire psychology network*. It computes agents' beliefs and desires and resulting emotions in the service of action prediction and explanation. All of the six task types identified by Schurz et al. [14] require these computations. The network apparently does not concern itself with the purely epistemic question of other people's differing views, an aspect shared only by the false belief tasks and vPT.

## Figures and Tables

**Table 1:** Predictions of overlap of vPT with theory of mind according to various proposals.

	Proposal	mPFC	Right TPJ/IPL	Left TPJ/IPL	TPJ/pSTS	IFG
(1)	Amodio & Frith [31]	Yes				
(2)	Gallagher et al. (2003)	(Yes)				
(3)	Schurz et al. [14]	Yes	Yes	Yes	Yes	
(4)	Van Overwalle [32]	No	Yes	Yes		
(5)	Gobbini et al. [33]		No	No	No	
(6)	Mar [34]		No	No		
(7)	Saxe et al. [35]					
	(a) Propositional content		(Yes)			
	(b) Belief & thoughts		No			
(8)	Perner & Leekam [36]			(Yes)	No	
(9)	C. D. Frith & U. Frith [37]				No	
(10)	Samson & Apperly [38]	possibly		Yes		Yes: right
*Observed*		*No*	*No*	*Yes*	*No*	*Yes: left*

Note: for predictions in parenthesis it is assumed that vPT tasks engage level 2 perspective taking processes.

**Table 2:** Examples of typical tasks entering the vPT meta-analysis by Schurz et al. [10].

Author	Img.	Experimental task	Control task
		*Level 1 visual perspective taking*	
Vogeley [47]	fMRI *n* = 11	You see a scene including an avatar and a number of objects. Indicate how many objects the avatar can see.	You see a scene including an avatar and a number of objects. Indicate how many objects you see.

		*Level 2 visual perspective taking*	
Aichhorn [9]	fMRI *n* = 18	You see a scene including an avatar and two objects. Indicate their relative spatial arrangement, for example, “block is in front of the pole” from the viewpoint of an avatar.	You see a scene including an avatar and two objects. Indicate their relative spatial arrangement, for example, “block is in front of the pole” from your own viewpoint.

		*Level 2 imagined viewer rotation*	
Zacks [48]	fMRI *n* = 16	You see an array of four objects. Imagine viewing the array from a different angle (i.e., imagine a self-rotation around the array). Indicate if a particular object is now on the left or right side of the array.	You see an array of four objects. Imagine that the array rotates along its vertical axis, while your own position remains the same. Indicate if a particular object is now on the left or right side of the array.

**Table 3:** Examples for each task-group from the theory of mind meta-analysis by Schurz et al. [14].

Author	Img.	Experimental task	Control task
		*False belief*	
Saxe [49]	fMRI *n* = 21	Read a short vignette involving a person holding a false belief. Answer a question about her belief. For example,* “John told Emily that he had a Porsche. Actually, his car is a Ford. Emily doesn't know anything about cars so she believed John. When Emily sees John's car, she thinks it is a …?” (Porsche or Ford).*	Read a false-photograph vignette. Answer a question concerning the outdated content in the photo. For example,* “A photograph was taken of an apple hanging on a tree branch. The film took half an hour to develop. In the meantime, a strong wind blew the apple to the ground. The developed photograph shows the apple on the…? (tree or ground).”*

		*Trait judgments*	
Mitchell [50]	fMRI *n* = 34	Read an adjective. Indicate whether it can be true for a hypothetical person. For example,* “ ‘nervous'…can it be true for ‘David?'?”*	Read an adjective. Indicate whether it can be true for an object. For example,* “ ‘sundried'…can it be true for ‘grape'?”*

		*Strategic games*	
Assaf [51]	fMRI *n* = 18	Play a “domino game” with a human opponent (you get feedback about her moves). You and your opponent hold some domino chips in your hands (undisclosed). On each turn, you must play out a domino chip with a particular number to get a game point. You play out your chips face-down (undisclosed), so you can pretend having the required number even if you have not. After you played out a chip, your opponent can decide whether or not to check the number on it *(simplified description)*.	Play a “domino game” with a computer.

		*Social animation*	
Castelli [52]	PET *N* = 6	Watch a video animation of two interacting triangles (e.g., *mother and child are playing*). Explain verbally what was happening (after fMRI).	Watch video animation of two randomly moving triangles. Explain verbally what was happening (after fMRI).

		*Mind in the eyes*	
Baron-Cohen [53]	fMRI *n* = 12	View photographs of eyes. Indicate which of two words (e.g., *concerned* versus *unconcerned*) describes the mental state of that person.	View photographs of eyes. Indicate if the person is male or female. See Baron-Cohen 1999.

		*Rational action*	
Brunet [54]	fMRI *n* = 8	View a cartoon story and predict what will happen based on intentions of a character (no false belief). Choose a logical story ending from several options shown in pictures. For example, *a prisoner is in his cell. First, he breaks the bars of his prison window. Then he walks to his bed. Participants must indicate what will happen next…the prisoner ties a rope from the sheets on his bed/the prisoner shouts out loud.*	View a cartoon story and predict what will happen based on physical causality. Choose a logical story ending from several options shown in pictures. For example, *a person is standing in front of a slide. A large ball is coming down this slide, heading towards the person standing there. Participants must indicate what will happen next…the ball is knocking over the person/the ball is resting on the ground and the person is standing next to it.*

**Table 4:** Summary of the conjunction analysis of visual perspective taking and different theory of mind tasks.

Hem	Label	False belief					Trait Judgment					Social animation						Mind in the Eye					Rational action			
		Cluster peak					Cluster peak					Cluster peak						Cluster peak					Cluster peak			
		*x*	*y*	*z*	*z*-val	*vx*	*x*	*y*	*z*	*z*-val	*vx*	*x*	*y*	*z*	*z*-val	*vx*	*x*	*y*	*z*	*z*-val	*vx*	*x*	*y*	*z*	*z*-val	*vx*
L	Mid. occipital	−38	−72	26	2.53	12																−48	−70	12	2.27	3
																						−48	−74	12	2.1	1
L	Mid. temporal	−44	−70	10	2.33	12	−42	−74	26	1.87	1															
L	TPJ/angular gyrus	−40	−60	36	2.79	27																				
		−36	−60	28	2.29	3																				
L	Precuneus cortex	−2	−56	46	2.76	71	−2	−54	40	2.17	5											−2	−60	40	2.42	61
R	Precuneus cortex	2	−56	44	2.66	52																4	−58	40	2.59	40
L	Precentral/mid. frontal gyrus																−46	6	32	3.45	405					
																	−36	8	44	2.39	3					
L	Inf. frontal gyrus											−48	14	26	2.86	12	−52	8	20	2.7	3					
L	Frontal operculum											−40	10	12	2.52	7	−42	12	0	2.77	99					
R	Frontal operculum																38	16	8	2.34	4					

Regions are reported from posterior to anterior. *Hem*: hemisphere of peak; *Label*: anatomical labelling corresponding to the cluster peak and subpeak (according to Harvard-Oxford cortical and subcortical structural atlases); *cluster peak*: cluster peaks are reported in Talairach space; *z-val*: maximum *Z* value; *vx*: cluster extent in voxel. All coordinates are reported in Talairach space. Strategic game task is not included in the table due to no overlapping region.
